# Stem Cell Transplantation for Muscular Dystrophy: The Challenge of Immune Response

**DOI:** 10.1155/2014/964010

**Published:** 2014-06-26

**Authors:** Sara Martina Maffioletti, Maddalena Noviello, Karen English, Francesco Saverio Tedesco

**Affiliations:** ^1^Department of Cell and Developmental Biology, University College London, London WC1E 6DE, UK; ^2^Experimental Hematology Unit, Division of Immunology, Transplantation and Infectious Diseases, San Raffaele Scientific Institute, 20132 Milan, Italy; ^3^Institute of Immunology, Department of Biology, National University of Ireland Maynooth, County Kildare, Ireland

## Abstract

Treating muscle disorders poses several challenges to the rapidly evolving field of regenerative medicine. Considerable progress has been made in isolating, characterizing, and expanding myogenic stem cells and, although we are now envisaging strategies to generate very large numbers of transplantable cells (e.g., by differentiating induced pluripotent stem cells), limitations directly linked to the interaction between transplanted cells and the host will continue to hamper a successful outcome. Among these limitations, host inflammatory and immune responses challenge the critical phases after cell delivery, including engraftment, migration, and differentiation. Therefore, it is key to study the mechanisms and dynamics that impair the efficacy of cell transplants in order to develop strategies that can ultimately improve the outcome of allogeneic and autologous stem cell therapies, in particular for severe disease such as muscular dystrophies. In this review we provide an overview of the main players and issues involved in this process and discuss potential approaches that might be beneficial for future regenerative therapies of skeletal muscle.

## 1. Introduction

Stem cell therapies hold promises for a plethora of conditions involving the loss or damage of resident tissue progenitors, including skeletal muscle. Skeletal muscle is the most abundant human tissue and its accessibility makes it a good candidate for protocols based upon the delivery of stem cells as a medicinal product. Disorders affecting skeletal muscle can be acute, such as trauma-related tissue damage or loss, and chronic, such as tissue wasting in muscular dystrophies, as typical of Duchenne muscular dystrophy (DMD), the most common paediatric inherited muscle disorder. DMD is an X-linked progressive and degenerative myopathy characterised by muscle wasting and weakness, which ultimately leads to loss of ambulation in puberty, cardiac and respiratory involvement, and premature death [1].

Different cell therapy strategies have been tested, in particular for chronic skeletal muscle disorders, using diverse types of cells with myogenic potential derived from muscle (e.g., satellite cells/myoblasts, muscle derived stem cells), vessels (e.g., pericytes and their progeny, mesoangioblasts), bone marrow, blood, or embryonic tissues, including, recently, induced pluripotent stem cells (reviewed in [2]). Some of these cells, such as mesoangioblasts, are currently completing clinical experimentation for DMD. However, the data obtained from this multitude of studies resulted in promising but suboptimal efficacy in restoring functional skeletal muscle tissue. Therefore, there is still no efficacious cell therapy-based treatment for muscle diseases. The reasons behind this are linked to challenges associated with the medicinal product (myogenic stem cells) and with the target tissue, the multinucleated, abundant, and widespread skeletal muscle [3]. General bottlenecks of cell therapies are represented by the availability of an adequate number of stem cells to transplant, which includes problems related to the harvesting from donors or from the same patient, genetic correction (in case of autologous transplant), maintenance of myogenic potential prior to transplantation, and large scale amplification in culture under appropriate conditions and by their compatibility with the host immune system. Specific hurdles related to skeletal muscle are due to some of the tissue's intrinsic features. First of all, skeletal muscle is the most abundant tissue in the human body (several kilograms per individual) and hence cell replacement strategies require high numbers of transplantable progenitors (several million per kilogram). Moreover, the administration route greatly influences the extent of grafting [4]. Indeed transplanted cells undergo a limited, although variable, migration from the site of injection that decreases the efficiency of the treatment. Intra-arterial delivery of the cells is an alternative, but it is limited to cells that have the ability to cross the vessel wall (such as pericyte-derived mesoangioblasts and CD133^+^ cells) [2]. This issue might be of minor relevance for the treatment of localized disorders but remains one of the most important to be overcome for the treatment of systemic muscle pathologies.

In addition to the aforementioned problems, a complex immune response further complicates and impairs the outcome of cell transplants. Data from myoblast transplantation studies indicate that 90% of donor cells are cleared within the first hour after transplantation by cell-mediated immune responses [5–7]. Moreover, muscles affected by chronic diseases are in a state of persistent inflammation and are characterized by an abundant infiltrate of immune cells that may hamper extensive grafting, proliferation, and differentiation of the transplanted stem cells into functional muscle tissue. The aim of this review is to give a general overview on the role of the immune system in the context of skeletal muscle regeneration focusing on the interaction of immune cells and transplanted stem cells in cell therapy strategies for muscular dystrophies. Inflammatory myopathies [8] represent another broad spectrum of muscle disorders with a predominant immunological aspect. Although in this type of disorder the immune system plays a primary role in provoking the muscle pathology, this will not be discussed here as it goes beyond the scope of this review.

## 2. Immune Response during Muscle Regeneration

**Figure 1:**
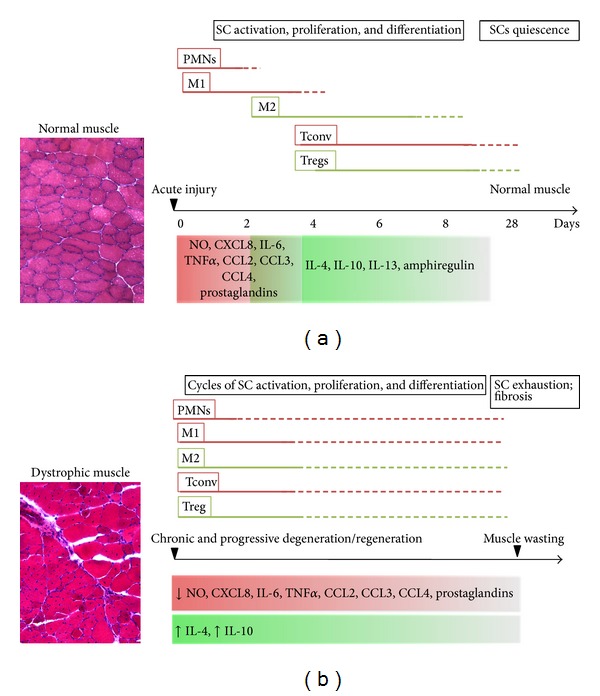
Dynamics of inflammation and muscle regeneration in acute and chronic injury. Acute muscle injury (a) triggers local release of chemoattractants that induce PMNs and M1 invasion into the damaged tissue. PMNs and M1 release an array of molecules (such as NO) that further amplify local inflammation, contributing to debris clearance and SC activation. This initial Th1-driven inflammation is later overcome by an anti-inflammatory response that coincides with a M1-to-M2 switch. By day 4 Tregs reach the site of injury, modulating Tconv expansion and activation and SC differentiation through amphiregulin release. M2 and Th2 cytokines reduce local inflammation and contribute to SCs differentiation, thus promoting the latest stages of muscle regeneration. Upon damage repair, SCs return to quiescence. Chronic muscle injuries (b) are characterised by persistent inflammation. Muscles feature infiltrates of PMNs, M1 together with M2, Tconv, and Treg; moreover, the simultaneous release of pro- and anti-inflammatory molecules promotes incomplete tissue regeneration and fibrosis. The SC pool undergoes depletion due to continuous rounds of activation and differentiation, resulting in terminal muscle wasting. SC: satellite cells; PMN: neutrophils; M1: M1 macrophages; M2: M2 macrophages; Tconv: CD4^+^ conventional T cell; Tregs: CD4^+^ Foxp3^+^ regulatory T cell; NO: nitric oxide; Red box: Th1-driven response; Green box; Th2-driven response.

Skeletal muscle originates from embryonic mesoderm and each muscle is composed of several muscle fibres (its functional unit). Each myofibre is a large syncytium containing numerous nuclei within the same cytoplasm [9]. The fibres' plasma membrane (also known as sarcolemma) is in tight contact with the satellite cells, the main resident stem cell population of skeletal muscle [10, 11]. The satellite cell niche is indeed localised between the basement membrane encircling each myofibre and the fibres' sarcolemma. Skeletal muscle has a conspicuous regenerative ability and relatively large injuries can be repaired in a few weeks. Upon activation satellite cells produce transit-amplifying progenitors called myoblasts (which will fuse with preexisting fibres or generate new fibres) and give also rise to stem cells able to maintain the pool of undifferentiated satellite cells for further rounds of regeneration (reviewed in [12]). This regeneration process is tightly orchestrated and entails the interplay of different cell types of muscle origin but also inflammatory and immune cells (Figure 1). Indeed the latter plays a very important role in all the stages of the process and alterations to any of the components impair the regenerative response.

### 2.1. Muscle Regeneration in Acute Injury

In skeletal muscle, acute injury either by myotoxin injection, freeze, crush, or exercise-related damage triggers a stereotypical response. Injury initiates an innate immune response characterized by proinflammatory cytokines. Soon after damage, a wave of neutrophils invades the area with a peak in their concentration at 24 hours followed by a rapid decrease [13]. Neutrophils release proinflammatory molecules (such as CXCL8 and interleukin-6 (IL-6)) that recruit macrophages into the tissue. Resident macrophages, present in the interstitial spaces of the epimysium and perimysium, play key roles especially in the first phases of acute injury [14]. Macrophages are rapidly activated and polarized towards a M1 inflammatory phenotype (“classically activated” macrophages). Neutrophils and M1 macrophages produce an array of molecules, including cytokines, chemokines, nitric oxide, and prostaglandins that sustain and amplify local inflammation [15].

Tumour necrosis factor *α* (TNF*α*) is the main proinflammatory cytokine present upon skeletal muscle damage [16]. It is initially released by degranulation of resident mast cells followed by infiltrating neutrophils and macrophages [17, 18]. Importantly, TNF*α* promotes activation and proliferation of satellite cells [19] while inhibiting their differentiation [20–22]. These effects are mediated by the activation of nuclear factor kappa B (NF-*κ*B) [23].

Chemokine (C-C motif) ligands 2, 3, and 4 (CCL2, CCL3, and CCL4) are chemoattractant molecules that play a significant part in muscle regeneration and their receptors, CCR2 and CCR5, are upregulated following skeletal muscle injury [24, 25]. Data obtained from knock-out mice showed that CCR2 is required for macrophage invasion of the injury site, with impaired regeneration in CCR2-null mice in parallel with a slowed revascularization of the injured area [26]. Moreover, satellite cells and myoblasts constitutively express CCR1, CCR2, CCR4, and CCR5 [27].

M1 macrophages express the inducible nitric oxide synthase (iNOS) and hence release nitric oxide (NO) in the injury site. Although NO can damage muscle cell membranes, it also facilitates tissue debris clearance by targeting them for phagocytosis [28]. In addition, oxidative stress caused by NO stimulates satellite cell proliferation [29], a process essential for muscle regeneration.

Other key molecules present in the inflammatory phase of muscle regeneration are prostaglandins. These signalling molecules are derived from the metabolism of arachidonic acid by cyclooxygenases (COX) and expression of COX-1 and COX-2 is indeed very high in injured muscles [30]. The effects of prostaglandins have been reported in all the stages of muscle regeneration, from satellite cells proliferation [31] to differentiation [32] and fusion [33].

The M1-driven tissue inflammation is gradually overcome by an anti-inflammatory response due to a switch in macrophage phenotype, from M1 to M2 (“alternatively activated”) [34]. M2-polarized macrophages are activated by anti-inflammatory Th2 cytokines such as IL-4, IL-10, and IL-13 that attenuate the inflammatory response through M1 deactivation [35]. Moreover, IL-4 and IL-10 act directly on muscle cells, inducing myogenin expression and subsequent differentiation and fusion [34, 36]. Hence, while M1 macrophages contribute to creating an inflammatory environment that helps clearing cell debris and activating satellite cells, M2 macrophages reduce inflammation and promote myogenic differentiation.

Although molecules are secreted by immune cells shape regeneration, the muscle is not a passive bystander. Indeed it releases a series of cytokines and chemokines collectively referred to as “myokines” [37] which also include IL-6, TNF*α*, and CCL2 [38, 39]. Aside from proinflammatory functions, IL-6 was demonstrated to have both an autocrine function on satellite cell proliferation and muscle hypertrophy [40] and paracrine effects when released into the circulation (on glucose metabolism and lipolysis) [41].

### 2.2. Regeneration in Chronic Muscle Disorders

While acute muscle injuries are characterized by a self-limiting physiological inflammatory reaction, chronic muscle conditions are generally associated with persistent inflammation. Recent data indicate that inflammation plays an active part in the pathology [42]. Chronic myopathies are a heterogeneous group of diseases characterized by progressive muscle wasting and include muscular dystrophies (e.g., DMD), which will be the focus of this review.

The important role of inflammation in muscular dystrophies is supported by the efficacy of corticosteroid treatments in improving muscle strength and function in the short term in patients [43]. Indeed muscular dystrophies are generally characterized by an infiltrate of inflammatory neutrophils and phagocytic M1 macrophages that produce inflammatory cytokines and NO, as in the case in acute injuries [44]. A hallmark of chronic muscle pathologies is the infiltration of M2 macrophages at early stages; this differs from acute muscle injury, where M2 macrophages invade the tissue only at later time points. M2 macrophages express the enzyme arginase, which shares its substrate (arginine) with iNOS expressed by M1 macrophages and this M1-M2 substrate competition decreases NO production [44]. M2 macrophages also induce a Th1-Th2 shift of cytokine production, with an increase of IL-4 and IL-10, which can deactivate M1 macrophages [35]. This Th2-dominated environment also induces the activation of eosinophils that promote muscle fibrosis through major basic protein-1-mediated processes [45]. Data obtained from mdx mice and DMD patients suggest that, besides the innate immune response, some degree of cellular immunity is also involved [46, 47]. DMD muscles are in fact characterized by the presence of infiltrating T cells expressing a highly conserved peptide in the hypervariable domain of the T-cell receptor, suggesting a breakdown in peripheral tolerance [46, 48]. Recently, dystrophin-specific T-cell responses have been described in a considerable proportion of DMD patients. Of interest, the incidence of such responses was lower in the cohort of patients receiving deflazacort (a steroid) than in untreated ones, suggesting that the modulation of cellular immunity may contribute to the beneficial effect of corticosteroid treatment [48].

Other chronic disorders characterised by an altered regenerative and inflammatory pattern in the affected muscles include, for example, the groups of facioscapulohumeral muscular dystrophy (FSHD) and the limb girdle muscular dystrophies (LGMDs). Although these disorders are caused by different genetic alterations, both FSHD and LGMD have been shown to present clear hallmarks of inflammation (e.g., in FSHD1A [49] and LGMD2B [50]). However, the relevance to the onset and progression of the pathology remains ambiguous.

## 3. Immunologically Relevant Molecules Expressed by Muscle Cells

In both physiological and pathological conditions, there is an active interplay between muscle cells and cells of the immune system. This interaction is made possible by a shared panel of soluble and transmembrane molecules that transduce signals and form functional synapses between the two cell types [51, 52]. Beyond the already discussed soluble factors (cytokines and chemokines) and their receptors, muscle expresses other immunologically relevant molecules.

Toll-like receptors (TLRs) are the principal activators of the innate immune response. TLRs are expressed on multiple cell types (such as dendritic cells and macrophages) and generally respond to “danger signals” (e.g., pathogens and damage associated molecules) triggering the production of inflammatory cytokines and chemokines.* In vitro* studies demonstrated that murine myotubes express TLR2, TLR4, TLR5, and TLR9 [53]. Notably, a study showed that TLR3 is expressed in muscle biopsies of patients with chronic myopathies and that TLR3 activation on human myoblasts triggers a downstream cascade leading to NF-*κ*B activation and ultimately IL-8 production [54].

*In vitro*, myoblasts constitutively express major histocompatibility complex I (MHC I), which is upregulated upon inflammatory stimuli, such as IFN*γ*. IFN*γ* also induces the expression of MHC II in muscle cells [55, 56].* In vivo*, muscle fibres do not express MHC I or MHC II under physiological conditions [57], but they are expressed at high levels in inflammatory muscle disorders [58]. Due to the inducible MHC I/II expression, muscle cells are considered to be nonprofessional antigen presenting cells (APCs) and thus have the capacity to trigger T-cell-mediated immune response. In this context, it was demonstrated that human muscle cells possess all the intracellular machinery required for antigen processing in the context of MHC I/II presentation [59, 60]. Moreover, presence of fibroblast growth factor (FGF) in dystrophic muscle [61] may on the one hand regulate proliferation of myogenic progenitors [62] but on the other hand lead to expression of the MHC class II receptor HLA-DR, as it has been identified in human mesenchymal stem cells [63]. Aside from MHC molecules, myoblasts can express a nonclassical MHC I molecule, human leukocyte antigen-G (HLA-G), under inflammatory conditions [64]. HLA-G is a molecule with very low polymorphism and it is generally considered tolerogenic because of its role in maternal-foetal tolerance [65] and graft acceptance [66].

Canonical costimulatory molecules, such as the CD80 and CD86, required together with MHC for T-cells activation, have not been identified on muscle cells [67, 68]. CD40-CD40 ligand interaction is another key signal required in both humoral and cellular immunity. CD40 is usually present on the membrane of APCs and CD40 ligand on activated CD4^+^/CD8^+^ T cells, where it transduces signals for their stimulation and expansion. CD40 is also found expressed in other cell types [69, 70], including muscle cells.* In vitro*, human myoblasts constitutively express CD40 and its levels increase upon IFN*γ* and TNF*α* stimulation [67]. In this cell type, CD40-CD40 ligand interaction leads, among other effects, to an increase of intracellular adhesion molecule 1 (ICAM-1) expression [71] and hence to interaction with T cells present in the muscle tissue.

Adhesion molecules have a pivotal function in allowing interaction of muscle cells with immune cells. During skeletal muscle inflammation, ICAM-1 is expressed by both the endothelium of the vessels and by the muscle fibres [51, 72, 73]. ICAM-1 interacts with leukocyte function-associated antigen 1 (LFA-1), an integrin, which is expressed on T cells. ICAM-1/LFA-1 interaction leads to T-cell recruitment into the inflamed tissue (via ICAM-1 expression on endothelial cells) facilitating myofibre cytotoxicity (through myofiber-CD8^+^ T-cell interaction) [74].

In addition, PD-1/PD-L1 binding is thought to play an important role in suppressing immune responses [75]. Interestingly, expression of programmed death ligand 1 (PD-L1), an immune-inhibitory molecule, is induced in mesoangioblasts stimulated with proinflammatory cytokines [76] and in muscle biopsies from patients with idiopathic inflammatory myopathies [77]. Based on these findings, these cells are more likely to perform crucial functions in limiting, rather than priming, a muscle-directed immune response in inflammatory settings.

## 4. Interaction between Immune Cells and Muscle Stem Cells:* In Vitro* Studies

A large body of data provides evidence for the important part played by immune cells in shaping the regenerative response following muscle damage/injury. We have already discussed the key cells involved in this process and their role in promoting repair; however, much less is understood about the interactions between immune cells and muscle stem cells. Studies have shown the capacity for macrophages and macrophage-conditioned medium to enhance myogenic precursor cell adhesion and migration* in vitro* [78–80]. Blocking studies revealed pivotal functions for TNF-*α* and high mobility group box 1 (HMGB1) protein likely derived from macrophages [79, 80].

Dendritic cells (DC, professional APCs) have been identified in inflammatory infiltrates in muscle biopsies and may play an essential role in direct activation of antigen specific T cells [81]. Coculture of DC with myoblasts leads to a semimature DC phenotype with reduced ability to promote T-cell activation and proliferation in a cell contact dependent manner [82]. This study provided one of the first observations that muscle progenitor cells have immune modulatory capacity and could potentially promote a tolerogenic environment.

The adaptive immune response also has a role in inflammatory muscle disorders. Following activation by the innate immune system, dystrophin reactive T cells have been identified in DMD patients [48]. As such, these T cells may pose a threat both to autologous gene-corrected cell therapies and also to allogeneic cellular therapies. One* in vitro* study has investigated the effects of T cells on human mesoangioblasts and vice versa [76]. Despite an increase in HLA molecules expressed by mesoangioblasts following stimulation with IFN-*γ*, these cells failed to induce T-cell proliferation* in vitro*. Mesoangioblasts expressed low or negligible levels of the costimulatory molecules CD40, CD80, and CD86; however, following stimulation with proinflammatory cytokines significant expression of the inhibitory molecule PD-L1 was observed. This suggested that mesoangioblasts are hypoimmunogenic [76]. This study also examined the effects of mesoangioblasts on T cells and their immunosuppressive capacity* in vitro*. Mesoangioblasts suppressed both CD4^+^ and CD8^+^ T-cell proliferation in a dose and time dependent manner but did not induce anergy in T cells. In addition, mesoangioblasts inhibited T-cell production of proinflammatory cytokines. The mechanisms of action identified an important role for IFN-*γ* and TNF-*α* in activation of mesoangioblast to become immunosuppressive followed by secretion of indoleamine 2,3-dioxygenase (IDO) and prostaglandin E2 (PGE-2) to inhibit T-cell proliferation [76]. Similar findings were obtained using induced pluripotent stem (iPS) cell-derived mesoangioblast-like cells [83]. In addition, mesoangioblasts show peculiar resistance to T-cell killing, although they are recognized and killed by allogeneic T cells in an inflammatory microenvironment or upon differentiation into myotubes [84].

Aside from the positive effects that muscle stem cells may have in promoting a tolerogenic environment, there are negative interactions between these different cell populations. As discussed previously, neutrophils and macrophages are required for clearance of cell debris among other functions. In addition, cytotoxic killing of muscle cells by neutrophils was also reported [85]. This study identified interactions between neutrophils and macrophages, which promote macrophage killing of muscle cells* in vitro*. Similarly, TLR3 stimulation of muscle cells* in vitro* leads to the upregulation of the activating receptor NKG2D and subsequent natural killer (NK) mediated lysis of muscle cells [54]. However, it may be possible to reduce the susceptibility of muscle stem cells to NK-mediated lysis. Indeed a recent study demonstrated that prestimulation with IFN-*γ* can significantly decrease the susceptibility of allogenic human mesenchymal stem cells to activated NK-mediated cytotoxicity* in vitro* [86]. Few studies have been carried out to investigate the interactions between these two cell populations highlighting the need for additional investigation in this area. Importantly,* in vivo* models of muscle degeneration may provide a better understanding of the interactions between muscle stem cells and immune cells and how best to facilitate successful engraftment and function of stem cells.

## 5. Studying Immune Response upon Muscle Stem Cell Transplantation in Preclinical Models

### 5.1. Animal Models of Muscular Dystrophies

Animal models are extremely useful to investigate the pathogenesis of muscular dystrophies, the contribution of inflammation and immune responses in muscle repair and to evaluate safety and efficacy of novel therapeutic strategies. In particular, several animal models were developed for muscular dystrophies, among which the most commonly used is the X-linked muscular dystrophy mouse (mdx) carrying nonsense mutation in exon 23 of the dystrophin gene [87]. For the preclinical validation of transplanted human myogenic progenitors, immunodeficient murine models have been particularly helpful to minimize xenoreactivity and to facilitate engraftment of human cells. Several immunodeficient mice modelling DMD are available, including the nude/mdx mice [88], lacking the T-cell compartment, the SCID-mdx mice [89], lacking both T and B cells, and the recently described NSG-mdx^4CV^ [90], and Rag2^−^IL2rb^−^DMD^−^ mice [91], in which the NK cell activity is also defective. Dystrophic and immunodeficient mice are also available for limb-girdle muscular dystrophies (LGMD), including the alpha-sarcoglycan-null/scid/beige mouse for LGMD2D (alpha-sarcoglycan deficiency) [92] and the SCID/BlAJ mouse for LGMD2B (dysferlin deficiency) [93]. The above mouse models have been used to transplant nonsyngeneic cells harbouring additional transgenes (e.g., GFP), and some of them were also shown to be good recipients for novel human pluripotent stem cell-based protocols for muscular dystrophies [92, 94].

Among large animal models, the Golden Retriever muscular dystrophy (GRMD) [95, 96] and the Beagle-based canine X-linked muscular dystrophy (CXMD [97]) models were used in preclinical studies to demonstrate safety and efficacy of stem cell-based approaches for muscular dystrophies [98–100]. These independent studies shared the systemic delivery of nonmyoblast myogenic stem cells of mesodermal/mesenchymal origin, that is, mesoangioblasts [98], mesenchymal stromal cells (MyoD-transduced) [99] and muscle stem cells [100]. Allogeneic (with immunosuppressive therapy) and autologous gene-corrected stem cells were tested and overall results showed low frequency of inflammatory infiltrates and absence of anti-dystrophin antibodies.

Very recently the generation and characterisation of dystrophin-deficient pigs have been reported [101]. Even if this model appears to be particularly severe in comparison with the human disease progression, it could offer an additional platform for future studies. Although there are no dystrophic nonhuman primates, the use of wild type strains to optimize the design of cell therapy approaches has been reported. Several immunosuppressive regimen and cell injection modalities were compared in nonhuman primates to improve myoblast transplantation [102].

### 5.2. Stem Cell Transplantation

During the last decade, limitations in myoblast transplantation (detailed in the next section) fostered the search for other transplantable myogenic progenitors [2]. Given the pathological role of inflammation and immune dysregulation in muscular dystrophies [103, 104] and the high risk of rejection documented after myoblasts transplantation, many groups tried to find a transplantable cell type which combines myogenic potential together with anti-inflammatory and immunomodulatory properties. To this end, bone marrow-derived mesenchymal stromal cells (MSC, reviewed in [105]) were utilised for muscle regeneration [88]. However, a subsequent study showed that MSC engraftment into dystrophin-deficient mice did not result in spontaneous differentiation into muscle fibres and in functional recovery [106].

Mesoangioblasts are pericyte-derived progenitors that can be isolated from adult muscles and are able to differentiate into muscle fibres* in vitro* and* in vivo* upon transplantation (reviewed in [2]). The finding that allogeneic transplantation of mouse mesoangioblasts into alpha-sarcoglycan null dystrophic mice gave rise to alpha-sarcoglycan positive muscle fibres suggested that these cells may have some degree of immune evasion [107]. The immunosuppressive properties of human mesoangioblasts have been described above [76] and recent* in vitro* observations indicated that their immune privileged phenotype can be partially reverted during inflammation or upon differentiation [84]. A similar mechanism might be responsible for the negative outcome observed in a study where the alloreactive response of MSCs led to donor graft rejection [108]. Thus, although immune privileged stem/progenitors have regenerative capacity useful to treat muscle disorders, the survival of allogeneic stem cell progeny* in vivo* may still require pharmacological immunomodulation.

In the autologous setting (e.g., gene therapy strategies), host cells are genetically manipulated to correct or replace the defective gene. Based on the promising results obtained in dystrophic animals [109], clinical trials based on AAV-mediated gene transfer in muscles were designed to treat patients affected by inherited muscle disorders. However, the development of cellular and humoral responses specific for vector components [110] and/or for the transgene [111] posed important limitations and triggered further research to solve this issue and develop new gene therapy vectors [2].

One possible solution is the use of regulatory T cells (Treg) [112]. For instance, expansion of antigen specific Tregs after vector-mediated gene transfer to the liver leads to the induction of tolerance to the transgene [113, 114]. Recently, it was demonstrated that a specific subtype of clonally expanded Treg cells (specifically Foxp3^+^ CD4^+^ with a restricted TCR repertoire) was enriched in muscle upon acute or chronic injury, facilitating a nonimmunological role that favours tissue repair [115]. The authors proposed that Treg cells act, at least in part, by regulating the infiltrating myeloid population and by stimulating satellite cell proliferation and differentiation via the secretion of the growth factor amphiregulin [115].

### 5.3. Limitations of Xenografts

As the field of regenerative gene and cell therapy progresses and transgene expression reaches the threshold required for clinical benefit, the immune response elicited in human muscle remains a challenging issue that needs to be addressed to enhance the efficacy of these promising therapeutic approaches. Unfortunately, immunodeficient mice still show limited engraftment and are not able to predict host immune responses. Thus, further studies are needed to clarify the mechanism underlying these reactions and to identify potential targets of immune intervention. Possible options might be transplantation in juvenile mice (where the muscle is less “primed” by inflammation) [92], neonatal desensitization [116], or evasion of macrophage killing [117, 118].

Although transplantation of different human stem cell populations in immunodeficient mice allows studying their safety and efficacy profile, this assay gives only suboptimal results, possibly because of variables other than the immune system regulating donor cell engraftment. Indeed, several species-specific mechanisms of survival, migration, and expansion and differentiation depend on the direct interaction with the host environment (e.g., integrins and other proteins of the extracellular matrix), which in the case of a xenotransplant will be significantly mismatched. Overall, experiments in small and large animals paved the way to the clinical translation of therapeutic strategies based upon the infusion of healthy donor myogenic cells. These and other studies are analysed in the next section.

## 6. From Preclinical Studies to Clinical Trials

Following the promising results observed in mdx mice [119], a number of clinical trials in the early 1990s tested allogeneic transplantation of myoblasts to treat muscular dystrophies (reviewed in [7, 12]). Unfortunately, the outcome was disappointing due to the limited or absent dystrophin expression and to the limited gain in muscle strength of treated patients [120]. The major limitations to the success of allogeneic myoblasts transplantation were the high early mortality rate and the limited migratory abilities of myoblasts upon transplantation, together with the host immune reaction. The group of Tremblay treated dystrophic patients with intramuscular injection of allogeneic myoblasts in the absence of immune suppression and documented acute rejection of the cells [121]. This group then reported both cellular and humoral alloreactive responses in rodents, with myoblast-injected muscles infiltrated by activated CD4^+^ and CD8^+^ T lymphocytes and myoblast-reactive antibodies detected in recipient sera [122]. Thus, specific immune responses against injected cells were demonstrated and claimed to explain, at least in part, the suboptimal therapeutic benefits, suggesting the need for immune suppression to avoid acute rejection. Several immunosuppressive agents were therefore tested for their ability to promote myoblast engraftment in preclinical models. Tacrolimus administration was found to adequately control immune reactions without affecting myoblast proliferation and differentiation capacity, both in mice and in nonhuman primates [102, 123].

Another bottleneck for myoblast engraftment in mice was the high mortality rate of the injected cells during the first three days after transplantation. The early loss of donor cells was explained on the one hand with the variable viability of the cell preparation and on the other with inflammation-mediated events. Neutrophils and macrophages infiltrate myoblast-injected muscles within a few hours and likely mediate early cell death before the development of adaptive immune responses [124]. Interestingly, a study on myoblast dynamics indicated that only a minority of injected cells showing stem-cell-like behaviour have the chance to survive long-term and exert regenerative capacity, suggesting that immune rejection is not the only limitation of myoblast-based therapy [125]. Furthermore, other studies excluded a role for innate immune-mediated rejection [126]. Additionally, ischaemic necrosis of implanted cells was also found to be an important hurdle to a successful graft [127]. The limited migratory ability of myoblasts required multiple injections in separated sites in nonhuman primates [102] and represents a further limitation for the treatment of muscular dystrophies affecting the majority of skeletal muscles, including the diaphragm.

Although high-density injections of allogeneic myoblasts under tacrolimus administration led to the development of new muscle fibres in DMD patients [128], the current consensus is that myoblasts transplantation can be the elective treatment only for localized forms of muscles diseases. Indeed a recent phase I/IIa clinical study trial reported some benefit using autologous myoblast transplantation in the cricopharyngeal muscles of 12 adult patients affected by oculopharyngeal muscular dystrophy (OPMD), an autosomal dominant genetic disease characterized by ptosis and dysphagia. Safety and tolerability were observed in all the patients, together with an improvement in the quality of life score. No functional degradation in swallowing was observed for 10/12 patients [129]. At variance with most autologous transplantation strategies, the above study did not require any genetic correction of the medicinal product, since it was possible to do a biopsy in several healthy muscles of the same patients. However, this would not be possible for most muscular dystrophies and a possible preexisting immunity against the vector, the mutated protein, or the newly introduced wild type epitopes needs to be taken into account, although this might not correlate directly with a negative outcome [48, 129–131].

The need to overcome the hurdles observed in myoblasts transplantation studies for DMD prompted several laboratories to identify alternative populations of myogenic cells with a better profile in terms of expansion, survival, and migration. Among these, CD133^+^ cells and mesoangioblasts have been tested clinically. The safety of autologous transplantation of muscle-derived CD133^+^ cells was tested in eight boys with DMD in a double-blinded phase I clinical trial and no adverse events were reported [132]. Future follow-up studies based upon genetically corrected CD133^+^ are expected. A first-in-man phase I/II clinical trial based upon intra-arterial infusion of donor HLA-matched mesoangioblasts in 5 DMD boys receiving tacrolimus as immunosuppressive therapy is currently approaching conclusion at San Raffaele Hospital (Milan, Italy; EudraCT number 2011-000176-33). Clinical, biochemical and functional progress of the disease were followed during the year preceding treatment and were validated with a cohort of ambulant DMD boys and healthy controls [133]. Safety is the primary objective of the study and preliminary results indicate that the treatment is relatively safe. Indeed no adverse events due to immune suppression were observed, with good control of the immune response in the patients (Cossu et al., unpublished results). This study also provides clinical proof-of-principle for transplantation and intravascular delivery of nonhaematopoietic cells in DMD. Improvement of mesoangioblast extravasation upon modulation of endothelial junctional proteins in dystrophic mice has been recently published [134] and additional strategies to translate mesoangioblast transplantation to autologous settings, based upon human artificial chromosomes [135], reversible cell immortalization (Benedetti et al., in preparation) and differentiation of iPS cells [92, 136] are currently under development.

## 7. Conclusions

Stem cell transplantation for muscle disorders has faced several hurdles since the first trials more than 20 years ago. Progress has been made and myogenic stem cells other than myoblasts have entered clinical experimentation. Nevertheless, understanding what are the key factors allowing stable cell engraftment still remains critical for the success of allogeneic or autologous transplants in inflamed muscles. Clear immunological characterisation of stem cells (particularly when derived from pluripotent stem cells) together with a deeper understanding of the relevance of preexisting reactive T cells are issues that are undergoing intense investigation. Moreover, it is necessary to take into account other complex matters such as the insurgence of immune responses against the restored protein (e.g., against dystrophin in DMD) or against viral elements (in gene therapy settings) that might appear at different times. All these points add an additional level of complexity to the analysis of immune responses in stem cell therapies for muscular dystrophies and might require interventions beyond immunosuppression, such as induction of immune tolerance. Models and strategies to improve the outcome of xenotransplantation in immunodeficient animals will also be required in order to develop assays powerful enough to assess safety and efficacy of different types of myogenic stem cells. Even in such a case, some of the information necessary for the refinement of complex therapies (such as those based upon stem cells) will inevitably be unpredictable. However, prompt bedside-to-bench studies will bring invaluable insights to the field and, hopefully, efficacious solutions.
